# Clinical Characteristics and Prognosis of Patients with End-Stage Hypertrophic Cardiomyopathy from a Tertiary Center Cohort: Systolic Dysfunction and Advanced Diastolic Dysfunction

**DOI:** 10.3390/diagnostics15091134

**Published:** 2025-04-29

**Authors:** Andreea Sorina Afana, Robert Daniel Adam, Sebastian Militaru, Sebastian Onciul, Oana Andrei, Adela Chirita Emandi, Maria Puiu, Constantin Militaru, Ruxandra Jurcut

**Affiliations:** 1 Expert Center for Genetic Cardiovascular Diseases, Emergency Institute for Cardiovascular Diseases, 258 Fundeni Street, 022328 Bucharest, Romania; andreea.afana@gmail.com (A.S.A.); robertdanieladam@gmail.com (R.D.A.); oanaandrei2000@yahoo.com (O.A.); 2 Emergency Clinical County Hospital Craiova, 1 Tabaci Street, 200642 Craiova, Romania; sbmilitaru@gmail.com (S.M.); cccmilitaru@yahoo.com (C.M.); 3 Cardiology Department, University of Medicine and Pharmacy Craiova, 2 Petru Rares Street, 200349 Craiova, Romania; 4 Cardiology Department, University of Medicine and Pharmacy “Carol Davila”, 8 Eroii Sanitari Blvd., 050474 Bucharest, Romania; sebastian.onciul@gmail.com; 5 Emerald Medical Center, 75 Nicolae G. Caramfil Street, 077190 Bucharest, Romania; 6 Emergency Clinical Hospital Floreasca, 8 Calea Floreasca, 014461 Bucharest, Romania; 7 Regional Center of Medical Genetics Timiș, Part of ERN ITHACA, Clinical Emergency Hospital for Children “Louis Țurcanu” Timisoara, 2 Doctor Iosif Nemoianu Street, 300011 Timisoara, Romania; adela.chirita@umft.ro; 8 Department of Microscopic Morphology, Genetics Discipline, Center of Genomic Medicine, University of Medicine and Pharmacy “Victor Babes” Timisoara, 2 Piata Eftimie Murgu Street, 300041 Timisoara, Romania; 9 Institute for Research and Development in Genomics, 37 Dionisie Lupu, 020021 Bucharest, Romania; maria.puiu@genomica.gov.ro

**Keywords:** hypertrophic cardiomyopathy, end-stage hypertrophic cardiomyopathy, burn-out hypertrophic cardiomyopathy

## Abstract

**Background**: Hypertrophic cardiomyopathy (HCM) is a genetic disorder marked by myocardial hypertrophy, leading to diastolic and systolic dysfunction and heart failure. Traditionally, the burn-out stage is defined by systolic dysfunction, but we propose expanding its definition to include advanced diastolic dysfunction. **Methods**: We retrospectively analyzed HCM patients (2004–2023) with either systolic dysfunction (left ventricular ejection fraction [LVEF] < 50%) or advanced diastolic dysfunction (preserved LVEF with left atrial enlargement and elevated filling pressures: E/A ≥ 2 or E/e′ ≥ 14). Both subgroups were included under the term “end-stage HCM” and compared to HCM controls with preserved LVEF and impaired relaxation. **Results**: Of 696 HCM patients, 94 had end-stage HCM (23 with systolic dysfunction, 71 with advanced diastolic dysfunction). Median age was 56.5 years, and 55.3% were male. End-stage HCM patients were more symptomatic at follow-up than controls (91.5% vs. 75.0%, *p*-value = 0.006), with higher rates of dyspnea and advanced heart failure (38.3% vs. 6.3%, *p*-value < 0.001). Advanced diastolic dysfunction was associated with a more symptomatic profile (*p*-value = 0.013) and a high annual mortality rate (2.34%, *p* = 0.014). Male sex, older age, lower LVEF, and higher E/A predicted systolic dysfunction. **Conclusions**: Advanced diastolic dysfunction represents an alternative progression pathway in burn-out HCM, requiring distinct management strategies alongside systolic dysfunction.

## 1. Introduction

Hypertrophic cardiomyopathy (HCM) is a genetic cardiac disorder characterized by increased left ventricular (LV) wall thickness, with or without right ventricular (RV) hypertrophy, or elevated myocardial mass that cannot be entirely attributed to abnormal loading conditions [1].

Myocardial hypertrophy stiffens the LV, reducing its compliance and distensibility, which impairs diastolic filling. As a result, end-diastolic pressure rises, leading to increased pulmonary venous pressure and contributing to heart failure symptoms [2]. Diastolic dysfunction with preserved ejection fraction accounts for nearly 50% of heart failure (HF) cases, leading to significant morbidity and mortality [3,4].

Traditionally, the end stage of HCM has been described as a phase of adverse LV remodeling, marked by systolic dysfunction and myocardial scarring [5,6,7]. Recent literature suggests that this definition of the HCM burn-out phase should be expanded to include patients with advanced diastolic dysfunction [8], as progressive myocardial impairment in HCM may lead not only to systolic failure but also to restrictive physiology, representing an alternative pathway to disease progression. Therefore, we believe it is important to provide a clearer understanding of the clinical findings, management strategies, and outcomes for this challenging patient subgroup. Moreover, with the development of HF therapeutic options, prediction and early identification of patients at risk of progressing towards end-stage HCM would be instrumental for planning timely intervention to halt this evolution.

The aim of our study was to identify the clinical characteristics and prognosis of patients with end-stage disease among a large cohort of HCM patients from a tertiary center, as well as to identify predictors of this evolution.

## 2. Materials and Methods

### 2.1. Study Population

This retrospective, single-center study included patients hospitalized between January 2004 and December 2023 with the diagnosis of HCM who progressed to the advanced heart failure phase, categorized into two subtypes:

(i) overt LV systolic dysfunction (left ventricular ejection fraction [LVEF] < 50%) (oLVSD) or
(ii) advanced (severe) diastolic dysfunction (LVEF ≥50% in association with E/A ≥ 2 or E/e′ ≥ 14 and indexed left atrial end-systolic volume [LAESVi] > 34 mL/m^2^) (sDD).

We propose that patients with advanced diastolic dysfunction exhibit a clinical course with advanced heart failure and high mortality comparable to those already experiencing systolic dysfunction. Therefore, we classified both groups under the term “burn-out HCM”.

Patients with HCM who had a preserved LVEF (≥50%), as well as no restrictive physiology, matched for age and sex, were selected from the remaining HCM cohort of the center to serve as the control group.

We excluded patients with incomplete or poor-quality echocardiographic images, missing baseline echocardiography data, or lack of clinical or echocardiographic follow-up, as well as those with phenocopies and coronary artery disease.

### 2.2. Methods

We collected data from electronic medical records, including demographic characteristics, family history, blood tests, genetic analysis (Invitae Hypertrophic Cardiomyopathy Panel (44 genes tested)—INVITAE laboratory, San Francisco, USA or Hypertrophic Cardiomyopathy Panel (92 genes tested)—Blueprint Genetics laboratory, Espoo, Finland or TruSightCardio Panel Illumina (174 genes tested)—Genomic Center of the University of Medicine and Pharmacy Victor Babeș Timișoara), discharge medications, electrocardiographic (ECG) and Holter monitoring results, echocardiographic assessments, and cardiovascular magnetic resonance (CMR) evaluations. These data were collected from the patients’ initial visit to our center and the most recent follow-up available. The study was conducted in accordance with the principles outlined in the Declaration of Helsinki and was approved by the local ethics committee.

#### 2.2.1. Serum Biomarkers

A complete blood count and basic biochemical screening were performed for all patients. Light chain amyloidosis was ruled out through serum protein electrophoresis and free light chain analysis. Cardiac biomarkers, including N-terminal prohormone of brain natriuretic peptide (NT-proBNP) and brain natriuretic peptide (BNP), were measured at the time of each visit.

#### 2.2.2. Electrocardiogram

Baseline 12-lead surface ECG recordings were analyzed to assess QRS voltage and duration, as well as the presence of pseudoinfarction patterns. Additionally, atrial fibrillation and conduction abnormalities were documented.

#### 2.2.3. Echocardiography

Standard transthoracic echocardiography (was performed following the European Association for Cardiovascular Imaging guidelines using the same ultrasound machine Vivid E95 (GE Vingmed Ultrasound, Horten, Norway). LV systolic function was assessed by calculating the LVEF using Simpson’s method in both four- and two-chamber views. Diastolic function was evaluated by analyzing LV diastolic filling patterns and the ratio of the E-wave to mitral annular diastolic velocity (e′ wave), according to current international recommendations [9].

### 2.3. Clinical Outcomes

The primary endpoint was all-cause death. The follow-up period was defined as the interval between the diagnosis of HCM and either the date of the last clinical follow-up or the date of death, whichever occurred first. Outcome data were prospectively collected from electronic health records through December 2023. The primary outcome was all-cause mortality.

### 2.4. Statistical Analysis

Continuous variables were expressed as mean ± standard deviation (SD) if their distribution was normal, or as median and interquartile range (IQR) otherwise. Categorical variables were shown as frequencies and percentages. Comparisons between two groups were conducted using Student’s *t*-test (for normally distributed continuous variables) or Mann–Whitney U-test (for non-normally distributed continuous variables), and Chi-squared test (for categorical variables). For comparisons involving more than two groups, one-way ANOVA (for normally distributed variables) or Kruskal–Wallis test (for non-normally distributed variables) was used, followed by post-hoc pairwise comparisons with Bonferroni correction. The Cox proportional hazards regression was employed to assess the predictors of progression to the burn-out phase with systolic dysfunction in the overall HCM population. Variables for inclusion in the multivariable analysis were selected a priori based on current literature and established risk factors associated with progression toward the burn-out phase of HCM. In addition, we included parameters characterizing diastolic function to explore their potential role in this context. Hazard ratio (HR) and 95% confidence interval (CI) were calculated. Event-free survival curves were computed using the Kaplan–Meier method, and differences between the curves were compared using the log-rank test. A two-tailed *p*-value < 0.05 was used for statistical significance. Statistical analysis was performed using R software, version R 4.4.0 (R Project for Statistical Computing).

## 3. Results

### 3.1. Patient Characteristics

**Figure 1:**
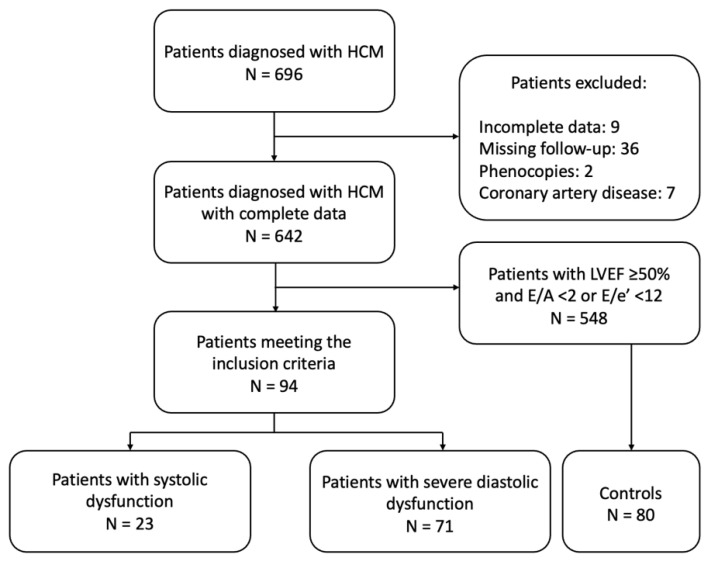
Flowchart of patient selection. Abbreviations: HCM: hypertrophic cardiomyopathy; LVEF: left ventricular ejection fraction.

Of the 696 consecutive HCM patients evaluated at our hospital, we excluded 9 patients due to incomplete data, 36 due to missing follow-up, 2 with further diagnosis of amyloidosis, and 7 with significant coronary artery disease (Figure 1). Among the remaining 642 patients, 94 (14.6%) were identified as being in the end-stage phase, including 23 (3.5%) with systolic dysfunction and 71 (11.1%) with advanced diastolic dysfunction. Among these 23 patients, 8 had systolic dysfunction at the initial evaluation, while 15 developed systolic dysfunction during follow-up. Of the 94 patients in the burn-out HCM group, only 80 were successfully matched to controls, as most available HCM patients with preserved LVEF and E/A < 2 and E/e′ < 14 did not meet the required age criteria for matching.

### 3.2. Characteristics of Burn-Out HCM Patients and Comparison with Controls

Table 1 presents the baseline characteristics of end-stage HCM patients and compares them with the controls. The HCM burn-out group consisted of patients with a median age of 56.5 years (IQR 47.0–63.0), with 55.3% being male. Proband status accounted for a significantly higher proportion of patients in the burn-out HCM group (93.6%) compared to the control group (78.8%) (*p*-value = 0.008). No significant differences were observed between the burn-out and control HCM groups in terms of age at diagnosis, age at atrial fibrillation diagnosis, age at death, family history of HCM or sudden cardiac death, and history of pregnancy. On average, 11.0 ± 8.6 years elapsed between HCM diagnosis and onset of systolic dysfunction.

Table 2 provides baseline and follow-up clinical, biological, ECG, and CMR characteristics of the entire HCM cohort, stratified into burn-out HCM and control HCM.

End-stage HCM patients were significantly more likely to be symptomatic at diagnosis (87.2% vs. 67.5%, *p*-value = 0.003), whereas syncope was more common in the control group (12.5% vs. 3.2%, *p*-value = 0.042). Diagnosis through routine checkups and family screening was more frequent in the control group (*p*-value = 0.035 and *p*-value = 0.009, respectively). No significant differences in genetic testing were observed between the groups; however, the low number of tested patients (47 [31.8%]) limited the assessment of the prognostic impact of variant status.

Regarding symptoms, burn-out HCM patients remained significantly more symptomatic at follow-up (91.5% vs. 75.0%, *p*-value = 0.006). Dyspnea was more prevalent in burn-out HCM both at baseline (78.7% vs. 52.5%, *p*-value < 0.001) and follow-up (87.2% vs. 63.7%, *p*-value = 0.001), with a higher incidence of advanced heart failure, with NYHA ≥ Class III at follow-up (38.3% vs. 6.3%, *p*-value < 0.001). Consistent with symptomatology, the end-stage HCM group exhibited higher BNP levels at both baseline (280 vs. 99 pg/mL, *p*-value < 0.001) and follow-up (316 vs. 177 pg/mL, *p*-value = 0.008), while N-terminal pro-brain natriuretic peptide levels were significantly elevated only at follow-up (3186 vs. 892 pg/mL, *p*-value < 0.001). The use of mineralocorticoid receptor antagonists (43.6% vs. 21.2%, *p*-value = 0.003) and loop diuretics (56.4% vs. 22.5%, *p*-value < 0.001) was significantly higher in the end-stage HCM group, suggesting a greater need for heart failure management. Nondihydropyridine calcium channel blocker use was also more common in burn-out HCM (8.51% vs. 1.25%, *p*-value = 0.040).

Regarding ECG findings, atrial fibrillation (37.2% vs. 6.25%, *p*-value < 0.001) and left bundle branch block (LBBB) (22.2% vs. 6.25%, *p*-value = 0.007) were more common in the end-stage HCM group. Also, they had a significantly higher HCM Risk Score at follow-up compared to controls (3.59% vs. 2.39%, *p*-value = 0.005). All patients received an implantable cardioverter-defibrillator (ICD) for primary prevention, except for one HCM control patient who experienced aborted sudden cardiac death and received an ICD for secondary prevention.

Due to the limited number of CMR exams performed (60 patients [34.7%]), no significant differences were detected between the two groups.

The analysis of the echocardiographic findings is presented in Table 3.

At follow-up, LV diameters were significantly larger in the end-stage group, indicating progressive LV dilatation (*p*-value = 0.005 for LV end-diastolic diameter [LVEDDi]), though LV volumes did not differ significantly between groups.

At baseline, the burn-out group had thicker walls (*p*-value = 0.003 for interventricular septum [IVS], *p*-value = 0.001 for posterior wall, and *p*-value = 0.036 for left ventricular maximum wall thickness [LVMWT]), but IVS thickness decreased over time (*p*-value = 0.030).

Systolic function was more impaired in the end-stage group, evidenced by a lower LVEF at follow-up (*p*-value < 0.001) with a significant decline over time (*p*-value = 0.011), as well as reduced septal and lateral s’ wave velocities at both baseline and follow-up (both *p*-value = 0.001). Left atrial end-systolic volume (LAESV) and LAESVi were significantly larger in the burn-out group at both time points (*p*-value < 0.001) with a significant increase over time (*p*-value = 0.046) for the non-indexed value. Although the difference between the burn-out and control groups did not reach statistical significance (*p*-value = 0.053), there is a noticeable trend toward larger LAESVi in the end-stage group.

Diastolic function was also worse in the burn-out group, demonstrated by a higher E/A ratio (*p*-value < 0.001 at baseline and *p*-value = 0.003 at follow-up) and elevated E/e′ ratio (*p*-value < 0.001 at both time points).

For the right ventricle, no significant differences were found in right ventricular maximum wall thickness, but systolic function was worse, with significantly lower RV S’ wave values at baseline and follow-up (both *p*-value < 0.001).

Mitral regurgitation was more severe in the end-stage group (*p*-value < 0.001), while tricuspid regurgitation was significantly worse in this group only at follow-up (*p*-value = 0.037).

### 3.3. Characteristics of End-Stage oLVSD HCM Subgroup Versus sDD HCM Subgroup

In the end-stage HCM group (Table S1), patients with systolic dysfunction tended to be younger at the time of death (56 vs. 71 years, *p*-value = 0.005), had a higher prevalence of relatives with SCD (55.5% vs. 31.0%, *p* = 0.032), and had a stronger family history of HCM (43.5% vs. 15.5%, *p*-value = 0.036) then patients with preserved LVEF. They also exhibited more pronounced ventricular remodeling at follow-up (*p*-value < 0.001 for LVEDDi, indexed left ventricular end-systolic volume [LVESVi]; *p*-value = 0.005 for indexed left ventricular end-diastolic volume [LVESDi]), thinner LV walls (LVWMT: 16 vs. 19 mm, *p*-value < 0.001), and more frequent electrical abnormalities, including a higher prevalence of LBBB (43.5% vs. 14.9%, *p*-value = 0.016) and prolonged QRS intervals (140 vs. 100 ms, *p*-value = 0.009). No major differences were observed in symptoms, biomarkers, or treatment, except for more severe dyspnea (73.9% vs. 26.8%, *p*-value < 0.001) and a greater use of mineralocorticoid receptor antagonists (73.9% vs. 33.8%, *p*-value = 0.003) among patients with systolic dysfunction.

### 3.4. Characteristics of End-Stage sDD HCM Subgroup Versus Controls

An additional analysis was performed to compare the end-stage HCM subgroup with systolic dysfunction to the control HCM group and the end-stage HCM subgroup with advanced diastolic dysfunction to the control HCM group (Table S1). Notably, significant differences from the control group persisted even after excluding patients with systolic dysfunction.

Furthermore, patients with end-stage HCM with advanced diastolic dysfunction were more likely to present with a symptomatic profile, including a higher prevalence of symptoms at diagnosis (85.9% vs. 67.5%, *p*-value = 0.042), dyspnea at baseline (78.9% vs. 52.5%, *p*-value = 0.004), and dyspnea at follow-up (84.5% vs. 63.7%, *p*-value = 0.010). They also exhibited higher biomarker levels, with BNP values at baseline (280 vs. 99 pg/mL, *p*-value < 0.001) and follow-up (310 vs. 177 pg/mL, *p*-value = 0.033), as well as a greater burden of atrial fibrillation (31.0% vs. 6.3%, *p*-value < 0.001).

From an echocardiographic perspective, patients with advanced diastolic dysfunction demonstrated more pronounced wall thickening (follow-up: 19.0 vs. 18.0 mm, *p*-value = 0.047), greater left atrial enlargement (LAESVi at baseline: 56.0 vs. 42.0 mL/m^2^; follow-up: 62.2 vs. 41.0 mL/m^2^; all *p*-value < 0.001), and more advanced diastolic dysfunction (E/A ratio at baseline: 1.3 vs. 0.9, *p*-value < 0.001; follow-up: 1.2 vs. 0.9, *p*-value = 0.017; E/e′ ratio at baseline: 18.6 vs. 10.0, *p*-value < 0.001; follow-up: 17.6 vs. 9.4, *p*-value < 0.001).

Although LVEF was not significantly different at baseline (64.0% vs. 63.0%, *p*-value = 0.568), it became significantly lower in the end-stage HCM group with advanced diastolic dysfunction at follow-up (60.0% vs. 63.5%, *p*-value = 0.006), even though it was preserved in all of the patients. The more pronounced systolic dysfunction in the end-stage HCM group with advanced diastolic dysfunction was further demonstrated by lower myocardial tissue velocities, including RV involvement (RV s’ wave: 12.6 vs. 14.1 cm/s, *p*-value = 0.006 at baseline; 12.0 vs. 13.0 cm/s, *p*-value = 0.036 at follow-up).

### 3.5. Primary Outcomes

After a median follow-up of 9.0 (6.0–16.0) years, the all-cause mortality rate in end-stage HCM patients was 26.6%, corresponding to an annual mortality rate of 3.38%. In the control group, a 6.25% mortality rate over 6.0 (4.0–10.2) years translated to an annual rate of 1.04% (*p*-value = 0.001). Mortality was even higher among the 23 patients with systolic dysfunction, reaching 43.5%, with an estimated annual death rate of 6.03% and a median age at death of 55.5 years (range: 41–65). Additionally, end-stage HCM patients with advanced diastolic dysfunction had a high annual all-cause mortality rate of 2.34%, which was significantly greater than that of the HCM control group (*p*-value = 0.014).

**Figure 2:**
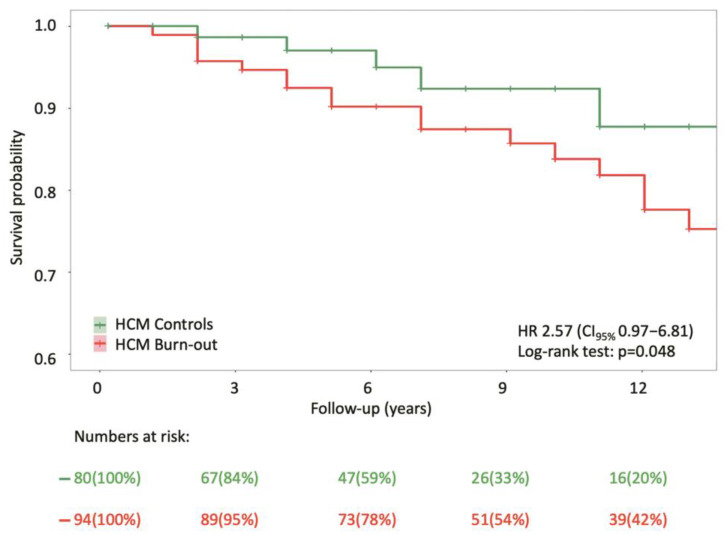
Kaplan–Meier survival curves for reaching all-cause death from the time of diagnosis of hypertrophic cardiomyopathy.

Based on the log-rank test (*p*-value = 0.048), Kaplan–Meier analysis (Figure 2) showed that patients with burn-out HCM (*N* = 94) were associated more strongly with all-cause death than controls (*N* = 80). However, the numbers at risk for the burn-out HCM group are 39 (42%), compared to 16 (20%) for the control group at 12 years. This discrepancy can be attributed to a higher number of censored individuals in the control group over the follow-up period.

### 3.6. Predictors of Burn-Out Phase in HCM

The univariate analysis (Table 4) identified as predictors for the burn-out phase to be male gender, resting dyspnea at diagnosis, dilated LV, poorer LVEF, baseline LVOT gradient, and high E/A ratio.

Males had a 4.5-fold higher risk (95% CI: 1.53–13.6, *p*-value = 0.006) of developing the burn-out phase. The presence of dyspnea at rest was strongly associated with an increased risk (HR: 9.51, 95% CI: 1.21–74.7, *p*-value = 0.032). Larger LV volumes (LVEDVi, *p*-value = 0.035; LVESVi, *p*-value < 0.001) at baseline, as well as a lower ejection fraction, were significant predictors of the burn-out phase. In contrast, LVOT obstruction at baseline was associated with a reduced risk (HR: 0.28, 95% CI: 0.09–0.84, *p*-value = 0.024). Atrial fibrillation showed a borderline significant association with the end stage (HR: 2.75, 95% CI: 1.00–7.55, *p*-value = 0.050). Additionally, a higher E/A ratio was linked to a 40% increased risk of burn-out (HR: 1.40, 95% CI: 1.02–1.92, *p*-value = 0.039).

After adjusting for covariates (Table 5), male sex remained a strong predictor (*p*-value = 0.002), along with older age at diagnosis (*p*-value = 0.003). Lower LVEF remained a highly significant predictor of the end stage (HR: 0.86, 95% CI: 0.79–0.94, *p*-value < 0.001), whereas LV volumes were no longer significant after adjustment. However, a higher E/A ratio remained independently associated with the burn-out phase (HR: 1.57, 95% CI: 1.15–2.12, *p*-value = 0.004).

## 4. Discussion

This study analyzed the predictor factors and prognosis of end-stage HCM in a Romanian cohort. The major findings are the following:

(i) in total, 3.5% of patients with HCM in a tertiary center’s cohort developed systolic dysfunction with LVEF < 50%, while 11.1% evolved advanced diastolic dysfunction;
(ii) with a higher mortality risk compared to controls, 26.6% of burn-out HCM patients experienced all-cause death after a median follow-up of 9.0 (6.0–16.0) years, with a mortality rate rising to 43.5% among those with systolic dysfunction;
(iii) burn-out HCM patients were significantly more symptomatic at follow-up, with a higher prevalence of dyspnea and advanced heart failure, requiring more intensive heart failure management, with higher use of diuretics and mineralocorticoid receptor antagonists;
(iv) male sex, older age at diagnosis, lower LVEF, and a higher E/A ratio were independent predictors of progression to the burn-out phase with systolic dysfunction;
(v) patients with burn-out HCM with advanced diastolic dysfunction exhibited a more symptomatic profile, higher biomarker levels, and a greater atrial fibrillation burden compared to controls.

The prevalence of systolic dysfunction was lower than in other reported cohorts [10,11], whereas Harris et al. observed a comparable rate [12]. In contrast, a recent cohort study by Marstrand et al. identified an even higher prevalence, reaching 8% [13]. For patients with systolic dysfunction, the estimated annual mortality rate was 6.03% in our cohort, with a total mortality rate of 43.5% over the entire follow-up period (median: 9.0 years). Harris et al. reported an almost double annual mortality rate of 11% in HCM patients with an LVEF < 50% [12]. Similarly, Rowin et al. found 17% all-cause mortality in this subgroup over a shorter follow-up of 5.8 ± 4.7 years [14]. Additionally, Maron et al. reported that patients with HCM and advanced heart failure may account for up to 60% of HCM-related deaths [15].

Most patients with burn-out HCM were males (55.3%), in concordance with other studies [16,17]. The likelihood of developing systolic dysfunction was 9.15 times higher in males than in females, consistent with previously published data [18]. Older age at diagnosis was more common in patients with end-stage HCM in the present study, in concordance with Marstrand et al. [13]. For each 1 year increase in age at diagnosis, the risk of developing the burn-out phase increases by 6%. In contrast, Biagini et al. reported that a younger age at diagnosis was associated with the progression to dilated-hypokinetic HCM [10].

Although sarcomeric variants are well known to be linked to the burn-out evolution of HCM [13], less than one-third of patients in our cohort underwent genetic testing, and the results did not show significant differences between groups.

As expected, heart failure symptoms were more prevalent in the burn-out HCM group, as evidenced by a higher incidence of dyspnea, elevated biomarker levels, and greater use of diuretics. These findings remained significant even in burn-out HCM patients with preserved LVEF but advanced diastolic dysfunction, underscoring the need for close monitoring in this population. Restrictive diastolic dysfunction has been established as an independent predictor of poor prognosis in HCM, even in the absence of systolic dysfunction, particularly in relation to heart failure outcomes [19,20]. Atrial fibrillation is closely corelated with LA enlargement [21]. LA dilatation and atrial fibrillation were more prevalent in the end-stage HCM group, even among patients with advanced diastolic dysfunction only. Diastolic dysfunction, driven by myocyte hypertrophy and disarray, along with reduced left ventricular compliance and elevated end-diastolic pressures, appears to be a key factor in the initiation and persistence of atrial fibrillation. At the same time, atrial fibrillation shortens left ventricular filling time, further exacerbating diastolic dysfunction, promoting left atrial dilation, and contributing to extensive cardiac remodeling [22]. A cohort study by Olivotto et al. demonstrated that HCM patients with atrial fibrillation had a significantly higher risk of heart failure-related mortality (OR 3.7, *p*-value < 0.002) [21].

Patients in the burn-out HCM group exhibited a thicker IVS at the initial evaluation, a finding that has also been observed in other cohorts [10]. In 1998, Maron et al. described the progression of hypertrophic cardiomyopathy to the burn-out stage, reporting an increase in LVEDD of 1 to 1.5 mm per year and a 20% reduction in LVMWT over 5–6 years [23]. Similarly, in our burn-out cohort, we observed a slight decrease in IVS thickness over time.

Patients with burn-out HCM exhibited similar rates of LVOT obstruction as the control group; however, when obstruction was present, the gradients at baseline were higher in the burn-out cohort. Maron et al. identified LVOT obstruction as an independent predictor of severe symptoms of heart failure [24]. Both obstructive and non-obstructive HCM cases can progress to end-stage disease, with advanced diastolic dysfunction playing a significant role in obstructive cases.

RV involvement is usually seen in 17% of HCM cases [25]. Similar to our findings, Sadr Ameli et al. reported a significantly lower S wave of the RV in the burn-out group [26].

Cardiac fibrosis is a well-established factor associated with burn-out HCM [14,27,28,29]. However, due to limited data, the presence of late gadolinium enhancement was not found to be significantly higher in our burn-out HCM cohort.

A Cox regression model was performed to identify predictors of developing burn-out HCM with systolic disfunction. In the model adjusted for sex, age at diagnosis, symptoms at diagnosis, echographic LVEF, indexed LA, and LV volumes and E/e′ ratio, the presence of an increase in E/A ratio was associated with an HR of 1.57 (95% CI: 1.15–2.12, *p*-value = 0.004) for developing systolic disfunction. Marstrand et al. identified increased left ventricular cavity size as a significant predictor of LV systolic dysfunction in HCM [13]. Although LVEDVi and LVESVi were associated with the burn-out phase in univariate Cox analysis, these findings were not confirmed by multivariate analysis. Although LAESVi was not independently linked to progression of diastolic dysfunction in our cohort, Yang et al. found that an increased LA volume index independently predicted cardiovascular events, excluding death (HR 1.44, 95% CI 1.12–1.83; *p*-value < 0.01) [30].

These findings emphasize the significance of evaluating diastolic function, as the end-stage HCM phenotype with advanced diastolic dysfunction is associated with poor prognosis in terms of mortality and morbidity, with a high incidence of heart failure. Our results align with those of an Italian cohort, further validating this observation [8]. LA enlargement and atrial fibrillation were also frequently observed in those without systolic impairment, reinforcing the bidirectional link between diastolic dysfunction and atrial remodeling. These findings highlight the potential impact of early rhythm surveillance and proactive atrial fibrillation management on long-term outcomes. This study sheds light on the burden and clinical trajectory of end-stage HCM within a Romanian cohort, emphasizing the importance of early identification, personalized management strategies, and comprehensive monitoring of both systolic and diastolic function to enable timely interventions and improve prognosis.

Given the prognostic importance of restrictive physiology, future trials should evaluate therapies aimed specifically at improving diastolic function in HCM, even in the absence of systolic impairment.

### Limitations

This study has several limitations. First, as a retrospective study, some examination results were incomplete. Moreover, as most deaths occurred outside the hospital, it was challenging to determine the exact causes of death. As a result, only all-cause mortality was included in the analysis. Also, the study population was drawn from a single center, which may limit the generalizability of the findings. However, our center serves as the primary referral center in the country, with patients being sent from various hospitals nationwide for further evaluation. Finally, genetic analysis and CMR data were not comprehensively assessed due to the low rate of these investigations.

## 5. Conclusions

While HCM with systolic dysfunction is a well-recognized condition associated with increased morbidity and mortality, it is equally important to acknowledge disease progression across all HCM phenotypes. Effective risk stratification and optimal management require physicians to be familiar with the full spectrum of clinical presentations. In particular, patients with HCM and advanced diastolic dysfunction form a distinct subgroup that often experiences progressive heart failure symptoms and should not be overlooked in clinical evaluation and treatment. Therefore, increased awareness and targeted therapeutic strategies are essential to improving outcomes for this population.

## Figures and Tables

**Table 1:** Baseline demographic characteristics of the entire HCM cohort (*N* = 174) and of the cohort stratified by HCM phenotypes: end-stage (*N* = 94) versus control (*N* = 80).

Variable	Overall (*N* = 174) ^1^	End-Stage HCM (*N* = 94) ^1^	Control HCM (*N* = 80) ^1^	*p*-Value ^2^
HCM end-stage phenotype:				
With oLVSD *, *n* (%)	-	23 (24.5%)	-	-
With sDD †, *n* (%)	-	71 (75.5%)	-	-
Demographic features:				
Male, *n* (%)	97 (55.7%)	52 (55.3%)	45 (56.2%)	1.000
Proband status, *n* (%)	151 (86.8%)	88 (93.6%)	63 (78.8%)	**0.008**
Age at baseline visit (years)	57.0 (46.2–63.8)	56.5 (47.0–63.0)	57.0 (45.5–64.2)	0.967
Age at diagnosis of HCM (years)	50.1 ± 15.2	49.5 ± 15.6	50.8 ± 14.9	0.560
Age at diagnosis of LV systolic dysfunction (years), *N* = 23	-	55.3 ± 12.5	-	-
Time from diagnosis of HCM to diagnosis of systolic dysfunction (years), *N* = 23	-	11.0 ± 8.6	-	-
Time from diagnosis of HCM to death (years), *N* = 30	7.0 (4.0–15.0)	9.5 (4.0–17.2)	6.0 (4.0–7.0)	0.271
Age at diagnosis of atrial fibrillation	56.9 ± 13.1	56.7 ± 13.0	57.4 ± 14.9	0.835
Age at death	65.0 (56.0–72.5)	66.0 (56.8–74.5)	60.0 (56.0–64.0)	0.248
History of pregnancy, *n* (% women)	48 (72.7%)	32 (71.1%)	16 (76.2%)	0.893
Family history:				
Family history of HCM, *n* (%)	41 (23.6%)	21 (22.3%)	20 (25.0%)	0.816
Family history of SCD, *n* (%)	45 (25.9%)	27 (28.7%)	18 (22.5%)	0.447
Age at SCD for relatives (years)	51.5 (30.8–60.0)	52.0 (33.5–60.0)	40.0 (30.0–54.0)	0.761
Age at death for relatives with confirmed HCM (years)	51.5 ± 21.4	45.7 ± 27.0	57.3 ± 13.8	0.339

^1^ Median (IQR), mean ± SD, *n* (%). ^2^ Mann–Whitney U test, *t*-test and Chi-squared test. Bold *p*-values indicate statistical significance (*p*-value < 0.05). Abbreviations: HCM: hypertrophic cardiomyopathy; SCD: sudden cardiac death. * Characterized by LVEF < 50%. † Characterized by E/A > 2 or E/e′ > 12, but FEVS ≥ 50%.

**Table 2:** Baseline and follow-up clinical, biological, ECG, and CMR characteristics of the entire HCM cohort (*N* = 174) and of the cohort stratified by HCM phenotypes: end-stage (*N* = 94) versus control (*N* = 80).

Variable	Overall (*N* = 174) ^1^	End-Stage HCM (*N* = 94) ^1^	Control HCM (*N* = 80) ^1^	*p*-Value ^2^
Clinical findings at diagnosis:				
Symptomatic at diagnosis, *n* (%)	136 (78.2%)	82 (87.2%)	54 (67.5%)	**0.003**
Exertional dyspnea at diagnosis, *n* (%)	61 (35.3%)	39 (41.9%)	22 (27.5%)	0.068
Resting dyspnea at diagnosis, *n* (%)	2 (1.2%)	2 (2.1%)	0 (0%)	0.500
Angina at diagnosis, *n* (%)	42 (24.3%)	26 (28.0%)	16 (20.0%)	0.299
Palpitations at diagnosis, *n* (%)	15 (8.6%)	9 (9.6%)	6 (7.5%)	0.830
Syncope at diagnosis, *n* (%)	13 (7.5%)	3 (3.2%)	10 (12.5%)	**0.042**
Routine checkup at diagnosis, *n* (%)	29 (16.7%)	10 (10.6%)	19 (23.8%)	**0.035**
Family screening at diagnosis, *n* (%)	6 (3.5%)	0 (0%)	6 (7.5%)	**0.009**
Genetic testing:				
Number of genetic tests, *n* (%)	47 (31.8%)	25 (26.6%)	22 (40.7%)	0.110
Negative genetic testing, *n* (%)	10/47 (21.3%)	6/25 (24.0%)	4/22 (18.2%)	0.730
MYBPC3 variant, *n* (%)	11/47 (23.4%)	4/25 (16.0%)	7/22 (31.8%)	0.351
MYH7 variant, *n* (%)	13/47 (27.7%)	8/25 (32.0%)	5/22 (22.7%)	0.702
Another HCM-related variant, *n* (%)	13/47 (27.7%)	7/25 (28.0%)	6/22 (27.3%)	1.000
P/LP variant, *n* (%)	33/47 (70.2%)	18/25 (72.0%)	15/22 (68.2%)	0.063
VUS, *n* (%)	13/47 (27.7%)	10/25 (40.0%)	3/22 (13.6%)	0.502
Clinical findings at baseline visit:				
Symptomatic at baseline visit, *n* (%)	143 (82.7%)	81 (86.2%)	62 (78.5%)	0.259
Dyspnea at baseline visit, *n* (%)	116 (66.7%)	74 (78.7%)	42 (52.5%)	**<0.001**
NYHA class at baseline, *n* (%)				0.190
Class I	62 (35.7%)	28 (28.8%)	34 (42.5%)	
Class II	90 (51.7%)	53 (56.4%)	37 (46.2%)	
Class III	20 (11.5%)	11 (11.7%)	9 (11.2%)	
Class IV	2 (1.2%)	2 (2.1%)	0 (0%)	
Dyspnea NYHA ≥ Class III at baseline, *n* (%)	22 (12.6%)	13 (13.8%)	9 (11.2%)	0.778
Palpitations at baseline visit, *n* (%)	40 (23.0%)	23 (24.5%)	17 (21.2%)	0.747
Syncope at baseline visit, *n* (%)	21 (12.1%)	12 (12.8%)	9 (11.2%)	
Angina at baseline visit, *n* (%)	72 (41.4%)	40 (42.6%)	32 (40.0%)	0.852
Aborted SCD at baseline visit, *n* (%)	1 (0.6%)	0 (0.0%)	1 (1.3%)	0.460
HCM SCD risk score at baseline (%)	2.6 (1.8–4.1)	2.7 (2.0–4.5)	2.5 (1.6–3.6)	0.117
Biomarkers at baseline visit:				
BNP at baseline visit (pg/mL)	192 (99.0–434)	280 (173–633)	99.0 (51.1–235)	**<0.001**
NT-proBNP at baseline visit (pg/mL)	1092 (269–2531)	1800 (838–3223)	658 (241–1612)	0.123
ECG features:				
Atrial fibrillation at follow-up visit, *n* (%)	40 (23.0%)	35 (37.2%)	5 (6.3%)	**<0.001**
Ventricular pacing at follow-up visit, *n* (%)	16 (9.20%)	11 (11.7%)	5 (6.25%)	0.328
PR duration follow-up (ms)	160 (140–186)	160 (140–180)	160 (140–190)	0.833
QRS duration follow-up (ms)	100 (90.0–130)	105 (90.0–138)	99.5 (89.5–113)	0.061
Presence of LBBB at follow-up visit, *n* (%)	25 (14.7%)	20 (22.2%)	5 (6.3%)	**0.007**
Presence of RBBB at follow-up visit, *n* (%)	16 (9.4%)	9 (9.9%)	7 (8.8%)	1.000
Negative T waves antero-lateral at follow-up visit, *n* (%)	117 (69.2%)	66 (74.2%)	51 (63.7%)	0.195
Negative T waves inferior at follow-up visit, *n* (%)	32 (18.9%)	17 (19.1%)	15 (18.8%)	1.000
QRS microvoltage at follow-up visit, *n* (%)	1 (0.6%)	1 (1.1%)	0 (0%)	1.000
Holter ECG monitoring:				
Number of Holter ECG monitoring studies, *n* (%)	152 (87.4%)	84 (89.4%)	68 (85.0%)	0.526
NSVT, *n* (%)	38/152 (25.0%)	22/84 (26.2%)	16/68 (23.5%)	0.760
STV, *n* (%)	4/152 (2.6%)	3/84 (3.6%)	1/68 (1.5%)	0.626
Frequent PVCs *, *n* (%)	11/152 (7.2%)	8/84 (9.5%)	3/68 (4.4%)	0.342
Atrial fibrillation, *n* (%)	38/152 (25.0%)	23/84 (27.4%)	15/68 (22.1%)	0.497
ICD interrogation:				
VT detected, *n* (%)	6 (17.1%)	3 (12.5%)	3 (27.3%)	0.352
ATP, *n* (%)	3 (8.57%)	1 (4.17%)	2 (18.2%)	0.227
Appropriate shock, *n* (%)	4 (11.4%)	3 (12.5%)	1 (9.0%)	1.000
CMR features:				
Patients with CMR studies, *n* (%)	60 (34.7%)	25 (26.9%)	35 (43.8%)	**0.030**
LGE presence, *n* (%)	46/60 (76.7%)	21/25 (84.0%)	25/35 (71.4%)	0.170
LVEF at CMR (%)	63.1 ± 12.0	59.7 ± 11.4	65.3 ± 12.0	0.109
Clinical findings at follow-up visit:				
Symptomatic at follow-up visit, *n* (%)	146 (83.9%)	86 (91.5%)	60 (75.0%)	**0.006**
Dyspnea at follow-up visit, *n* (%)	133 (76.4%)	82 (87.2%)	51 (63.7%)	**0.001**
NYHA class at follow-up, *n* (%)				**<0.001**
Class I	49 (28.2%)	19 (20.2%)	30 (37.5%)	
Class II	82 (47.1%)	37 (39.4%)	45 (56.2%)	
Class III	32 (18.4%)	27 (28.7%)	5 (6.25%)	
Class IV	11 (6.32%)	11 (11.7%)	0 (0%)	
Dyspnea NYHA ≥ Class III, *n* (%)	41 (23.6%)	36 (38.3%)	5 (6.3%)	**<0.001**
Palpitations at follow-up visit, *n* (%)	30 (17.2%)	17 (18.1%)	13 (16.2%)	0.906
Syncope at follow-up visit, *n* (%)	22 (12.6%)	13 (13.8%)	9 (11.2%)	0.778
Angina at follow-up visit, *n* (%)	42 (24.1%)	17 (18.1%)	25 (31.2%)	0.065
Aborted SCD at follow-up visit, *n* (%)	0 (0.0%)	0 (0.0%)	0 (0.0%)	-
HCM SCD risk score at follow-up visit (%)	3.0 (1.8–5.3)	3.6 (2.2–6.0)	2.4 (1.6–4.3)	**0.005**
Biomarkers at follow-up visit:				
BNP at follow-up visit (pg/mL)	254 (129–589)	316 (180–622)	177 (80.1–358)	**0.008**
NT-proBNP at follow-up visit (pg/mL)	1104 (634–3079)	3186 (1871–7767)	892 (575–1096)	**<0.001**
Management and treatment:				
ICD, *n* (%)	36 (20.7%)	25 (26.6%)	11 (13.8%)	0.058
Pacemaker, *n* (%)	16 (9.2%)	11 (11.7%)	5 (6.3%)	0.328
CRT-P, *n* (%)	2 (1.16%)	2 (2.13%)	0 (0%)	0.501
CRT-D, *n* (%)	2 (1.15%)	2 (2.13%)	0 (0%)	0.500
History of septal myectomy, *n* (%)	7 (4.0%)	5 (5.3%)	2 (2.5%)	0.454
History of alcohol septal ablation, *n* (%)	4 (2.3%)	1 (1.1%)	3 (3.8%)	0.335
ACEI or sartan, *n* (%)	96 (55.2%)	50 (53.2%)	46 (57.5%)	0.677
Mineralocorticoid receptor antagonist, *n* (%)	58 (33.3%)	41 (43.6%)	17 (21.2%)	**0.003**
Loop diuretic, *n* (%)	71 (40.8%)	53 (56.4%)	18 (22.5%)	**<0.001**
Beta-blocker, *n* (%)	158 (90.8%)	84 (89.4%)	74 (92.5%)	0.652
Nondihydropyridine calcium channel blocker, *n* (%)	9 (5.2%)	8 (8.5%)	1 (1.3%)	**0.040**
Outcomes:				
All-cause death, *n* (%)	30 (17.2%)	25 (26.6%)	5 (6.3%)	**0.001**

^1^ Median (IQR), mean ± SD, *n* (%). ^2^ Mann–Whitney U test, *t*-test and Chi-squared test. Bold *p*-values indicate statistical significance (*p*-value < 0.05). * Defined by more than 20% of all QRS complexes on standard 24-h Holter monitoring. Abbreviations: ACEI: angiotensin-converting-enzyme inhibitors; ATP: anti-tachycardia pacing; BNP: brain natriuretic peptide; CMR: cardiovascular magnetic resonance; CRT-D: cardiac resynchronization therapy defibrillator; CRT-P: cardiac resynchronization therapy pacemaker; HCM: hypertrophic cardiomyopathy; ICD: implantable cardioverter-defibrillators; LP: likely pathogenic; LBBB: left bundle branch block; LGE: late gadolinium enhancement; LVEF: left ventricular ejection fraction; NSVT: nonsustained ventricular tachycardia; NT-proBNP: N-terminal pro-brain natriuretic peptide; NYHA: New York Heart Association; P: pathogenic; PVCs: premature ventricular contractions; RBBB: right bundle branch block; SCD: sudden cardiac death; SVT: sustained ventricular tachycardia; VT: ventricular tachycardia; VUS: variant of unknown significance.

**Table 3:** Analysis of echocardiographic findings at baseline and follow-up: end-stage (*N* = 94) versus control (*N* = 80).

	End-Stage HCM (*N* = 94)			Control HCM (*N* = 80)					
Variable	Baseline	Follow-Up	Change/Years	Baseline	Follow-Up	Change/Years	*p*-Value for Baseline	*p*-Value for Follow-Up	*p*-Value for Change
LVEDDi (mm/m^2^)	24.1 (22.0;26.8)	24.0 (21.8;27.0)	0.00 (−0.43;0.32)	23.1 (20.8;24.8)	22.6 (21.0;24.2)	−0.03 (−0.43;0.49)	**0.020**	**0.005**	0.897
LVEDVi (mL/m^2^)	48.8 ± 15.6	47.8 (36.0;58.2)	0.20 (−2.00;1.00)	48.0 ± 14.6	45.0 (35.4;55.5)	0.26 (−2.75;1.70)	0.827	0.346	0.752
LVESVi (mL/m^2^)	16.0 (10.6;20.60)	17.9 (11.8;28.5)	0.28 (−1.56;1.27)	15.3 (10.8;20.6)	16.0 (11.8;20.0)	−0.11 (−0.81;0.71)	0.949	0.178	0.627
LVEF (%)	63.5 (60.0;65.0)	60.0 (50.0;64.8)	−0.61 (−1.67;0.21)	63.0 (60.0;70.0)	63.5 (60.0;66.2)	0.00 (−0.58;0.80)	0.094	**<0.001**	**0.011**
IVS (mm)	19.0 (16.0;22.0)	18.0 (16.0;20.0)	−0.10 (−0.50;0.10)	17.0 (15.0;20.0)	17.0 (15.0;20.0)	0.00 (−0.22;0.25)	**0.003**	0.110	**0.030**
PW (mm)	13.0 (11.0;15.0)	13.0 (11.0;15.0)	0.00 (−0.54;0.18)	12.0 (10.8;13.0)	11.0 (10.0;13.0)	0.00 (−0.14;0.33)	**0.001**	**0.001**	0.158
LVMWT (mm)	20.0 (16.2;23.0)	18.0 (16.0;21.0)	−0.11 (−0.44;0.19)	18.0 (16.0;21.0)	18.0 (16.0;20.0)	0.00 (−0.33;0.25)	**0.036**	0.698	0.181
Apical hypertrophy, n (%)	6 (6.38%)	8 (8.51%)	-	12 (15.0%)	16 (20.0%)	-	0.107	0.049	-
LVOT obstruction *, n (%)	43 (45.7%)	28 (29.8%)	-	36 (45.0%)	30 (37.5%)	-	1.000	0.361	-
LVOT gradient † (mmHg)	88.0 (65.0;100)	85.0 (61.5;116)	−0.12 (−3.25;5.00)	71.5 (39.8;92.2)	64.0 (50.2;80.0)	−0.82 (−5.30;2.00)	**0.047**	**0.036**	0.620
Septal s’ wave (cm/s)	5.50 (4.00;6.00)	4.50 (4.00;6.00)	−0.03 (−0.25;0.02)	7.00 (6.00;8.00)	6.00 (5.30;7.70)	−0.08 (−0.29;0.09)	**<0.001**	**<0.001**	0.644
Lateral s’ wave (cm/s)	6.00 (5.00;7.00)	5.44 ± 1.50	−0.04 (−0.25;0.00)	7.80 (6.43;9.00)	7.18 ± 1.75	−0.11 (−0.33;0.11)	**<0.001**	**<0.001**	0.805
LAESV (mL)	103 (84.8;136)	124 (89.5;160)	1.77 (−0.80;4.00)	81.0 (60.2;96.0)	79.0 (64.0;100)	−0.06 (−2.50;2.30)	**<0.001**	**<0.001**	**0.046**
LAESVi (mL/m^2^)	56.0 (46.0;72.2)	65.7 (50.5;84.8)	0.84 (−0.40;2.00)	42.0 (31.2;48.2)	41.0 (34.5;51.5)	0.03 (−1.11;1.19)	**<0.001**	**<0.001**	0.053
E/A ratio	1.28 (0.91;2.20)	1.15 (0.76;2.04)	−0.01 (−0.18;0.06)	0.90 (0.75;1.20)	0.88 (0.71;1.17)	−0.01 (−0.05;0.02)	**<0.001**	**0.003**	0.690
E/e′ ratio	18.0 (15.0;22.0)	17.0 (12.2;20.8)	−0.08 (−1.21;0.36)	10.0 (8.00;11.4)	9.40 (7.70;11.0)	−0.03 (−0.37;0.20)	**<0.001**	**<0.001**	0.411
RVMWT (mm)	7.00 (6.00;8.00)	7.00 (6.00;8.00)	0.00 (0.00;0.16)	6.00 (5.00;7.00)	6.00 (5.00;7.00)	0.00 (−0.11;0.17)	0.083	0.051	0.251
RV s’ wave (cm/s)	12.3 ± 2.81	11.0 (8.00;13.0)	−0.09 (−0.49;0.10)	14.1 ± 2.54	13.0 (11.0;13.8)	−0.23 (−0.78;0.00)	**<0.001**	**<0.001**	0.171
*Severity of MR, n* (*%*):							**<0.001**	**<0.001**	-
No regurgitation	2 (2.20%)	1 (1.06%)		6 (7.50%)	6 (7.50%)				
Mild regurgitation	38 (41.8%)	44 (46.8%)		56 (70.0%)	53 (66.2%)				
Moderate regurgitation	27 (29.7%)	25 (26.6%)		13 (16.2%)	16 (20.0%)				
Moderate to severe regurgitation	19 (20.9%)	18 (19.1%)		5 (6.25%)	5 (6.25%)				
Severe regurgitation	5 (5.49%)	6 (6.38%)		0 (0.00%)	0 (0.00%)				
*Severity of TR, n* (*%*):							0.057	**0.037**	-
No regurgitation	8 (9.09%)	6 (6.45%)		12 (15.0%)	4 (5.00%)				
Mild regurgitation	55 (62.5%)	57 (61.3%)		59 (73.8%)	65 (81.2%)				
Moderate regurgitation	17 (19.3%)	21 (22.6%)		6 (7.50%)	9 (11.2%)				
Severe regurgitation	6 (6.82%)	5 (5.38%)		3 (3.75%)	2 (2.50%)				
Massive/torrential regurgitation	2 (2.27%)	4 (4.30%)		0 (0.00%)	0 (0.00%)				

Bold *p*-values indicate statistical significance (*p*-value < 0.05). * Defined by LVOT gradient at rest ≥30 mmHg. † Considering only obstructive cases, excluding those with gradients below 30 mmHg. Abbreviations: HCM: hypertrophic cardiomyopathy; IVS: interventricular septum; LAESV: left atrial end-systolic volume; LAESVi: indexed left atrial end-systolic volume; LVEDDi: indexed left ventricular end-diastolic diameter; LVEDVi: indexed left ventricular end-diastolic volume; LVEF: left ventricular ejection fraction; LVESVi: indexed left ventricular end-systolic volume; LVMWT: left ventricular maximum wall thickness; LVOT: left ventricular outflow tract; MR: mitral regurgitation; PW: posterior wall; RVMWT: right ventricular maximum wall thickness; TR: tricuspid regurgitation.

**Table 4:** Baseline predictors of evolution towards end-stage phase with systolic disfunction on univariate analysis in the overall HCM population (*N* = 174).

Characteristic	HR ^1^	95% CI ^1^	*p*-Value
Age at baseline visit (years)	0.98	0.95, 1.01	0.281
Male	4.56	1.53, 13.6	**0.006**
Proband status	0.68	0.16, 2.92	0.602
Family history of HCM	1.46	0.61, 3.50	0.393
Family history of SCD	1.59	0.67, 3.75	0.293
Symptomatic at diagnosis	2.08	0.48, 9.00	0.325
Exertional dyspnea at diagnosis	1.86	0.79, 4.37	0.156
Resting dyspnea at diagnosis	9.51	1.21, 74.7	**0.032**
Angina at diagnosis	0.89	0.29, 2.69	0.833
Palpitations at diagnosis	0.93	0.21, 3.99	0.917
Syncope at diagnosis	0.00	0.00, Inf	0.997
Routine checkup at diagnosis	0.93	0.27, 3.17	0.905
Family screening at diagnosis	0.00	0.00, Inf	0.998
Age at diagnosis of HCM	1.03	1.00, 1.07	0.060
Negative genetic testing	1.28	0.25, 6.61	0.769
MYBPC3 variant	1.55	0.36, 6.70	0.557
MYH7 variant	1.19	0.27, 5.13	0.818
P/LP variant	0.53	0.17, 1.67	0.279
VUS	1.20	0.35, 4.04	0.771
Symptomatic at baseline visit	0.52	0.19, 1.44	0.208
Dyspnea at baseline visit	1.91	0.70, 5.19	0.204
Dyspnea NYHA ≥ 3 at baseline	0.63	0.15, 2.74	0.539
Palpitations at baseline visit	1.23	0.51, 2.98	0.640
Syncope at baseline visit	0.21	0.03, 1.58	0.130
Angina at baseline visit	0.66	0.25, 1.69	0.383
Aborted SCD at baseline visit	0.00	0.00, Inf	0.998
HCM risk score at baseline (%)	0.91	0.74, 1.13	0.392
BNP at baseline visit (pg/mL)	1.00	1.00, 1.00	0.616
NT-proBNP at baseline visit (pg/mL)	1.00	1.00, 1.00	0.846
CK at baseline visit (U/L)	1.00	0.99, 1.01	0.984
CK-MB at baseline visit (U/L)	1.01	0.94, 1.07	0.866
Atrial fibrillation at baseline visit	1.06	0.31, 3.64	0.925
Ventricular pacing at baseline visit	0.83	0.10, 6.91	0.864
PR duration at baseline visit (ms)	1.00	0.99, 1.02	0.575
QRS duration at baseline visit (ms)	1.00	0.98, 1.02	0.905
Presence of LBBB at baseline visit	3.01	0.84, 10.8	0.091
Presence of RBBB at baseline visit	2.41	0.54, 10.8	0.250
Negative T waves antero-lateral at baseline visit	0.54	0.22, 1.35	0.190
Negative T waves inferior at baseline visit	1.83	0.51, 6.61	0.358
NSVT	0.86	0.31, 2.37	0.772
STV	0.00	0.00, Inf	0.998
Frequent PVCs *	1.46	0.41, 5.22	0.557
Atrial fibrillation	2.75	1.00, 7.55	0.050
Age at diagnosis of atrial fibrillation (years)	1.00	0.94, 1.05	0.896
VT detection	3.32	0.67, 16.5	0.143
ATP	1.72	0.19, 15.2	0.628
Appropriate shock	1.91	0.34, 10.6	0.461
LVEDDi baseline (mm/m^2^)	1.07	0.99, 1.16	0.105
LVEDVi baseline (mL/m^2^)	1.06	1.00, 1.11	**0.035**
LVESVi baseline (mL/m^2^)	1.16	1.08, 1.25	**<0.001**
LVEF baseline (%)	0.84	0.78, 0.91	**<0.001**
IVS baseline (mm)	1.02	0.94, 1.12	0.578
PW baseline (mm)	1.00	0.90, 1.11	0.967
LVMWT baseline (mm)	1.00	0.91, 1.09	0.939
Apical hypertrophy at baseline visit	1.37	0.39, 4.74	0.622
LVOT obstruction ^†^ at baseline visit	0.28	0.09, 0.84	**0.024**
LVOT gradient baseline (mmHg)	0.99	0.97, 1.02	0.493
Septal s’ wave baseline (cm/s)	0.85	0.57, 1.27	0.431
Lateral s’ wave baseline (cm/s)	0.77	0.51, 1.15	0.194
LAESV baseline (mL)	1.00	0.99, 1.01	0.954
LAESVi baseline (mL/m^2^)	1.00	0.97, 1.03	0.831
E/A ratio baseline	1.40	1.02, 1.92	**0.039**
E/e′ ratio baseline	0.94	0.84, 1.05	0.284
RVMWT baseline (mm)	1.08	0.70, 1.67	0.722
Tricuspid s’ wave baseline (cm/s)	0.90	0.75, 1.07	0.238
LGE presence (*N* = 60)	1.46	0.17, 12.7	0.729
CMR LVEF (%) (*N* = 60)	0.91	0.82, 1.00	0.050
ICD	1.18	0.49, 2.87	0.712
Pacemaker	1.70	0.62, 4.70	0.303
CRT-P	0.81	0.10, 6.69	0.844
CRT-D	1.80	0.40, 8.16	0.444
Septal myectomy	0.00	0.00, Inf	0.997
Alcohol septal ablation	0.00	0.00, Inf	0.998

^1^ HR = hazard ratio, CI = confidence interval. Bold *p*-values indicate statistical significance (*p*-value < 0.05). * Defined by more than 20% of all QRS complexes on standard 24 h Holter monitoring. ^†^ Defined by LVOT gradient at rest ≥ 30 mmHg. Abbreviations: same as Table 2.

**Table 5:** Baseline predictors of evolution towards end-stage phase with systolic disfunction on multivariate analysis in overall HCM population (*N* = 174).

Characteristic	HR ^1^	95% CI ^1^	*p*-Value
Male	9.15	2.26, 37.1	**0.002**
Symptoms at diagnosis	1.99	0.41, 9.57	0.393
Age at diagnosis of HCM	1.06	1.02, 1.11	**0.003**
LVEDVi baseline (mL/m^2^)	0.92	0.83, 1.03	0.162
LVESVi baseline (mL/m^2^)	1.15	0.98, 1.35	0.085
LVEF baseline (%)	0.86	0.79, 0.94	**<0.001**
LAESVi baseline (mL/m^2^)	0.99	0.95, 1.02	0.431
E/e′ ratio baseline	0.96	0.85, 1.10	0.575
E/A ratio baseline	1.57	1.15, 2.12	**0.004**

^1^ HR = hazard ratio, CI = confidence interval. Bold *p*-values indicate statistical significance (*p*-value < 0.05). Abbreviations: LAESVi: indexed left atrial end-systolic volume; LVEDVi: indexed left ventricular end-diastolic volume; LVEF: left ventricular ejection fraction; LVESVi: indexed left ventricular end-systolic volume.

## Data Availability

The raw data supporting the conclusions of this article will be made available by the authors on request.

## Supplementary Materials

The following supporting information can be downloaded at: https://www.mdpi.com/article/10.3390/diagnostics15091134/s1, Table S1. Baseline and follow-up clinical, biological, ECG, echographic, CMR characteristics of the end-stage HCM cohort with systolic dysfunction (*N* = 23) versus advanced diastolic dysfunction (*N* = 71) versus control HCM (*N* = 80) and their comparison.

## Abbreviations

The following abbreviations are used in this manuscript:

ATP	Anti-tachycardia pacing
BNP	Brain natriuretic peptide
CI	Confidence interval
CK	Creatine kinase
CMR	Cardiovascular magnetic resonance
CRT-D	Cardiac resynchronization therapy defibrillator
CRT-P	Cardiac resynchronization therapy pacemaker
HCM	Hypertrophic cardiomyopathy
ICD	Implantable cardioverter-defibrillator
IVS	Interventricular septum
LP	Likely pathogenic
LAESV	Left atrial end-systolic volume
LAESVi	Indexed left atrial end-systolic volume
LBBB	Left bundle branch block
LGE	Late gadolinium enhancement
LVEDDi	Indexed left ventricular end-diastolic diameter
LVEDVi	Indexed left ventricular end-diastolic volume
LVEF	Left ventricular ejection fraction
LVESVi	Indexed left ventricular end-systolic volume
LVMWT	Left ventricular maximum wall thickness
LVOT	Left ventricular outflow tract
MR	Mitral regurgitation
NSVT	Nonsustained ventricular tachycardia
NT-proBNP	N-terminal pro-brain natriuretic peptide
NYHA	New York Heart Association
P	Pathogenic
PVCs	Premature ventricular contractions
PW	Posterior wall
RBBB	Right bundle branch block
RVMWT	Right ventricular maximum wall thickness
SCD	Sudden cardiac death
SVT	Sustained ventricular tachycardia
TR	Tricuspid regurgitation
VT	Ventricular tachycardia
VUS	Variant of unknown significance
