# Supplementary material for: Clinical Characteristics and Prognosis of Patients with End-Stage Hypertrophic Cardiomyopathy from a Tertiary Center Cohort: Systolic Dysfunction and Advanced Diastolic Dysfunction
Source: Diagnostics (Basel). 2025 Apr 29;15(9):1134. doi: 10.3390/diagnostics15091134 (PMC12071236; doi:10.3390/diagnostics15091134)
Supplement: Supplementary file 1 [file diagnostics-15-01134-s001.zip › diagnostics-3602699-supplementary.pdf]

Supplemental file

Table S1. Baseline and follow-up clinical, biological, ECG, echographic, CMR characteristics of the end-stage HCM cohort with systolic dysfunction (N=23) versus advanced diastolic dysfunction (N=71) versus control HCM (N=80) and their comparison.

Variable	End-stage HCM with oLVSD (N=23)	End-stage HCM with sDD (N=71)	Control HCM (N=80)	p-value overall	p-value oLVSD vs sDD	p-value oLVSD vs control	p-value sDD vs control
**Demographic features:**							
Male, n (%)	18 (78.3%)	34 (47.9%)	45 (56.2%)	**0.039**	0.063	0.144	0.388
Proband status, n (%)	21 (91.3%)	67 (94.4%)	63 (78.8%)	**0.013**	0.632	0.345	**0.034**
Age at baseline visit (years)	52.0 (43.5-57.5)	58.0 (48.0-64.0)	57.0 (45.5-64.2)	0.181	0.175	0.269	0.494
Age at diagnosis of HCM	44.3±15.1	51.2±15.4	50.8±14.9	0.145	0.145	0.166	0.990
Age at diagnosis of atrial fibrillation (years)	58.0 (51.5-59.5)	58.0 (57.0-59.5)	58.0 (58.0-58.0)	0.670	0.713	0.713	0.843
Age at death (years)	54.7±9.3	71.1±9.1	59.0±10.7	**0.001**	**0.001**	0.687	**0.049**
History of pregnancy, n (%)	5 (83.3%)	27 (69.2%)	16 (76.2%)	0.770	1.000	1.000	1.000
Family History:							
Family history of HCM, n (%)	10 (43.5%)	11 (15.5%)	20 (25.0%)	**0.021**	**0.036**	0.214	0.214
Family history of SCD, n (%)	9 (39.1%)	18 (25.4%)	18 (22.5%)	0.273	0.473	0.473	0.826
Age at SCD for relatives (years)	31.0 (22.0-42.0)	55.0 (50.2-60.0)	40.0 (30.0-54.0)	**0.038**	**0.032**	0.229	0.229

Variable	End-stage HCM with oLVSD (N=23)	End-stage HCM with sDD (N=71)	Control HCM (N=80)	p-value overall	p-value oLVSD vs sDD	p-value oLVSD vs control	p-value sDD vs control
Age at death for relatives with confirmed HCM (years)	41.2±26.4	73.0	57.3±13.8	0.248	0.358	0.369	0.760
**Clinical findings at diagnosis:**							
Symptoms at diagnosis, n (%)	21 (91.3%)	61 (85.9%)	54 (67.5%)	**0.006**	0.724	0.069	**0.042**
Exertional dyspnea at diagnosis, n (%)	13 (56.5%)	26 (37.1%)	22 (27.5%)	**0.034**	0.247	0.058	0.277
Rest dyspnea at diagnosis, n (%)	1 (4.4%)	1 (1.4%)	0 (0%)	0.125	0.470	0.470	0.470
Angina at diagnosis, n (%)	4 (17.4%)	22 (31.4%)	16 (20.0%)	0.189	0.452	1.000	0.452
Palpitations at diagnosis, n (%)	2 (8.7%)	7 (9.9%)	6 (7.5%)	0.931	1.000	1.000	1.000
Syncope at diagnosis, n (%)	0 (0%)	3 (4.2%)	10 (12.5%)	0.065	1.000	0.193	0.193
Routine checkup at diagnosis, n (%)	3 (13.0%)	7 (9.86%)	19 (23.8%)	0.074	0.702	0.584	0.124
Family screening at diagnosis, n (%)	0 (0%)	0 (0%)	6 (7.50%)	**0.038**	-	0.333	0.060
**Genetic testing:**							

Variable	End-stage HCM with oLVSD (N=23)	End-stage HCM with sDD (N=71)	Control HCM (N=80)	p-value overall	p-value oLVSD vs sDD	p-value oLVSD vs control	p-value sDD vs control
Number of genetic tests, n (%)	9 (39.1%)	16 (22.5%)	22 (40.7%)	0.068	0.294	1.000	0.138
Negative genetic testing, n (%)	2 (22.2%)	4 (25.0%)	4 (18.2%)	0.897	1.000	1.000	1.000
MYBPC3 variant, n (%)	3 (33.3%)	1 (6.25%)	7 (31.8%)	0.131	0.174	1.000	0.174
MYH7 variant, n (%)	4 (44.4%)	4 (25.0%)	5 (22.7%)	0.483	0.591	0.591	1.000
Another variant, n (%)	0 (0%)	7 (43.8%)	6 (27.3%)	0.068	0.080	0.217	0.477
P/LP variant, n (%)	7 (53.8%)	11 (52.4%)	15 (83.3%)	0.095	1.000	0.171	0.171
VUS, n (%)	4 (30.8%)	6 (28.6%)	3 (17.6%)	0.719	1.000	0.998	0.998
**Clinical findings at baseline visit:**							
Symptoms at baseline visit, n (%)	18 (78.3%)	63 (88.7%)	62 (78.5%)	0.200	0.442	1.000	0.431
Dyspnea at baseline visit, n (%)	18 (78.3%)	56 (78.9%)	42 (52.5%)	**0.001**	1.000	0.074	**0.004**
Dyspnea NYHA≥3 at baseline, n (%)	2 (8.70%)	11 (15.5%)	9 (11.2%)	0.664	0.897	1.000	0.897
Palpitations at baseline visit, n (%)	9 (39.1%)	14 (19.7%)	17 (21.2%)	0.139	0.213	0.213	0.975
Syncope at baseline visit, n (%)	1 (4.35%)	11 (15.5%)	9 (11.2%)	0.401	0.598	0.598	0.598

Variable	End-stage HCM with oLVSD (N=23)	End-stage HCM with sDD (N=71)	Control HCM (N=80)	p-value overall	p-value oLVSD vs sDD	p-value oLVSD vs control	p-value sDD vs control
Angina at baseline visit, n (%)	7 (30.4%)	33 (46.5%)	32 (40.0%)	0.375	0.555	0.555	0.555
Aborted SCD at baseline visit, n (%)	0 (0%)	0 (0%)	1 (1.3%)	1.000	-	1.000	1.000
HCM risk score at baseline visit (%)	2.9 (1.9-4.6)	2.7 (2.0-4.4)	2.5 (1.6-3.6)	0.288	0.876	0.556	0.384
**Biomarkers at baseline visit:**							
BNP at baseline visit (ppg/ml)	360 (59.2-919)	280 (174-631)	99.0 (51.1-235)	**<0.001**	0.789	0.505	**<0.001**
NT-proBNP at baseline visit (pg/ml)	1360 (812-1908)	1800 (1272-3879)	658 (241-1612)	0.225	0.837	0.900	0.238
**ECG features:**							
Atrial fibrillation at follow-up visit, n (%)	13 (56.5%)	22 (31.0%)	5 (6.3%)	**<0.001**	0.051	**<0.001**	**<0.001**
Ventricular pacing at follow-up visit, n (%)	7 (30.4%)	4 (5.6%)	5 (6.3%)	**0.004**	**0.007**	**0.007**	1.000
PR duration follow-up (ms)	150 (110-180)	162 (141-180)	160 (140-190)	0.521	0.413	0.413	0.898
QRS duration follow-up (ms)	140 (100-160)	100 (90.0-125)	99.5 (89.5-113)	**0.004**	**0.009**	**0.004**	0.446
Presence of LBBB at follow-up visit, n (%)	10 (43.5%)	10 (14.9%)	5 (6.25%)	**<0.001**	**0.016**	**<0.001**	0.145

Variable	End-stage HCM with oLVSD (N=23)	End-stage HCM with sDD (N=71)	Control HCM (N=80)	p-value overall	p-value oLVSD vs sDD	p-value oLVSD vs control	p-value sDD vs control
Presence of RBBB at follow-up visit, n (%)	2 (8.7%)	7 (10.3%)	7 (8.8%)	0.935	1.000	1.000	1.000
Negative T waves antero-lateral at follow-up visit, n (%)	15 (71.4%)	51 (75.0%)	51 (63.7%)	0.327	0.967	0.967	0.585
Negative T waves inferior at follow-up visit, n (%)	5 (23.8%)	12 (17.6%)	15 (18.8%)	0.822	1.000	1.000	1.000
QRS microvoltage at follow-up visit, n (%)	1 (4.4%)	0 (0%)	0 (0%)	0.133	0.247	0.247	-
**Holter ECG monitoring:**							
Number of Holter ECG monitoring studies, n (%)	19 (82.6%)	65 (91.5%)	68 (85.0%)	0.339	0.485	0.751	0.485
NSVT, n (%)	7 (36.8%)	15 (22.4%)	16 (22.2%)	0.397	0.357	0.357	1.000
STV, n (%)	0 (0%)	3 (4.5%)	1 (1.4%)	0.614	1.000	1.000	1.000
Frequent PVCs*, n (%)	3 (15.8%)	5 (7.5%)	3 (4.2%)	0.187	0.482	0.309	0.482
Atrial fibrillation, n (%)	7 (36.8%)	16 (23.9%)	15 (20.8%)	0.333	0.607	0.607	0.820
**ICD interrogation:**							
VT detected, n (%)	3 (33.3%)	0 (0%)	3 (27.3%)	**0.041**	0.095	1.000	0.095
ATP, n (%)	1 (11.1%)	0 (0%)	2 (18.2%)	0.244	0.562	1.000	0.508
Appropriate shock, n (%)	2 (22.2%)	1 (6.7%)	1 (9.1%)	0.660	0.849	0.849	1.000

Variable	End-stage HCM with oLVSD (N=23)	End-stage HCM with sDD (N=71)	Control HCM (N=80)	p-value overall	p-value oLVSD vs sDD	p-value oLVSD vs control	p-value sDD vs control
**Echocardiographic features at baseline visit:**							
LVEDDi baseline (mm/m2)	27.5 (22.6-28.4)	23.9 (21.3-25.4)	23.1 (20.8-24.8)	**0.001**	**0.004**	**0.001**	0.226
LVEDVi baseline (ml/m2)	58.9±12.9	46.3±15.3	48.0±14.6	0.074	0.061	0.118	0.867
LVESVi baseline (ml/m2)	29.0 (25.2-31.1)	14.5 (9.18-18.2)	15.3 (10.8-20.6)	**0.006**	**0.005**	**0.010**	0.332
LVEF baseline (%)	60.0 (50.0-65.0)	64.0 (60.0-65.0)	63.0 (60.0-70.0)	**0.007**	**0.012**	**0.005**	0.568
IVS baseline (mm)	18.0 (15.5-22.0)	19.0 (16.0-22.0)	17.0 (15.0-20.0))	**0.009**	0.432	0.355	**0.006**
PW baseline (mm)	11.0 (10.0-12.5)	14.0 (12.0-15.5)	12.0 (10.8-13.0)	**<0.001**	**0.011**	0.374	**<0.001**
LVMWT baseline (mm)	19.0 (16.0-23.0)	20.0 (17.0-23.0)	18.0 (16.0-21.0)	0.087	0.563	0.563	0.074
Apical hypertrophy at baseline visit, n (%)	3 (13.0%)	3 (4.23%)	12 (15.0%)	0.060	0.232	1.000	0.158
LVOT obstruction† at baseline visit, n (%):	4 (17.4%)	39 (54.9%)	36 (45.0%)	**0.007**	**0.011**	**0.047**	0.291
LVOT gradient‡ baseline (mmHg)	69.0 (57.8-87.0)	88.0 (65.5-100)	71.5 (39.8-92.2)	0.108	0.553	0.685	0.123
Septal s′ wave baseline (cm/s)	5.0 (4.5-6.0)	5.8 (4.0-6.1)	7.0 (6.0-8.0)	**<0.001**	0.547	**0.002**	**<0.001**
Lateral s′ wave baseline (cm/s)	6.0 (4.3-6.8)	6.0 (5.1-7.0)	7.8 (6.4-9.0)	**<0.001**	0.281	**0.004**	**<0.001**
LAESV baseline (ml)	108 (87.0-132)	103 (84.5-137)	81.0 (60.2-96.0)	**<0.001**	0.764	**0.002**	**<0.001**

Variable	End-stage HCM with oLVSD (N=23)	End-stage HCM with sDD (N=71)	Control HCM (N=80)	p-value overall	p-value oLVSD vs sDD	p-value oLVSD vs control	p-value sDD vs control
LAESVi baseline (ml/m2)	56.4 (43.5-77.9)	56.0 (46.8-70.0)	42.0 (31.2-48.2)	**<0.001**	0.922	**0.003**	**<0.001**
E/A ratio baseline	1.1 (0.7-2.3)	1.3 (0.9-2.2)	0.9 (0.8-1.2)	**<0.001**	0.849	0.128	**<0.001**
E/e′ ratio baseline	13.5 (10.0-18.0)	18.6 (15.8-22.7)	10.0 (8.0-11.4)	**<0.001**	**0.003**	**0.008**	**<0.001**
RVMWT baseline (mm)	7.0 (6.0-8.0)	7.0 (6.0-8.0)	6.0 (5.0-7.0)	0.222	0.895	0.486	0.297
RV s′ wave baseline (cm/s)	11.2±3.8	12.6±2.5	14.1±2.5	**<0.001**	0.214	**0.001**	**0.006**
Severity of MR at baseline visit, n (%):				-	0.682	0.104	**<0.001**
No regurgitation	1 (5.0%)	1 (1.4%)	6 (7.5%)				
Mild regurgitation	9 (45.0%)	29 (40.8%)	56 (70.0%)				
Moderate regurgitation	6 (30.0%)	21 (29.6%)	13 (16.2%)				
Moderate to severe regurgitation	4 (20.0%)	15 (21.1%)	5 (6.3%)				
Severe regurgitation	0 (0%)	5 (7.0%)	0 (0%)				
Severity of TR at baseline visit, n (%):				0.133	0.765	0.695	0.095
No regurgitation	3 (16.7%)	5 (7.14%)	12 (15.0%)				
Mild regurgitation	11 (61.1%)	44 (62.9%)	59 (73.8%)				
Moderate regurgitation	3 (16.7%)	14 (20.0%)	6 (7.5%)				

Variable	End-stage HCM with oLVSD (N=23)	End-stage HCM with sDD (N=71)	Control HCM (N=80)	p-value overall	p-value oLVSD vs sDD	p-value oLVSD vs control	p-value sDD vs control
Severe regurgitation	1 (5.6%)	5 (7.1%)	3 (3.8%)				
Massive/ torrential regurgitation	0 (0%)	2 (2.9%)	0 (0%)				
**CMR features:**							
CMR available:	7 (31.8%)	18 (25.4%)	35 (43.8%)	0.058	0.747	0.669	0.085
LGE presence:	6 (85.7%)	15 (93.8%)	25 (73.5%)	0.272	0.660	0.660	0.414
CMR LVEF (%)	47.0 (9.41)	64.2 (8.21)	65.3 (12.0)	**0.004**	**0.010**	**0.003**	0.948
**Echocardiographic features at follow-up visit:**							
LVEDDi follow-up (mm/m2)	28.0 (27.0-30.7)	23.6 (21.4-25.5)	22.6 (21.0-24.2)	**<0.001**	**<0.001**	**<0.001**	0.177
LVEDVi follow-up (ml/m2)	58.0 (50.8-68.4)	41.3 (35.8-51.0)	45.0 (35.4-55.5)	**0.004**	**0.005**	**0.005**	0.651
LVESVi follow-up (ml/m2)	37.0 (26.1-45.0)	13.9 (10.7-18.0)	16.0 (11.8-20.0)	**<0.001**	**<0.001**	**<0.001**	0.230
LVEF follow-up (%)	37.0 (31.0-44.5)	60.0 (60.0-65.0)	63.5 (60.0-66.2)	**<0.001**	**<0.001**	**<0.001**	**0.006**
IVS follow-up (mm)	16.0 (13.2-18.0)	19.0 (16.5-21.5)	17.0 (15.0-20.0)	**<0.001**	**<0.001**	0.082	**0.008**
PW follow-up (mm)	12.0 (10.2-13.8)	13.0 (12.0-15.0)	11.0 (10.0-13.0)	**0.001**	0.072	0.636	**0.001**
LVMWT follow-up (mm)	16.0 (13.8-18.0)	19.0 (17.0-21.5)	18.0 (16.0-20.0)	**<0.001**	**<0.001**	**0.002**	**0.047**
Apical hypertrophy at follow-up visit, n (%)	3 (13.0%)	5 (7.0%)	16 (20.0%)	0.070	0.554	0.554	0.118

Variable	End-stage HCM with oLVSD (N=23)	End-stage HCM with sDD (N=71)	Control HCM (N=80)	p-value overall	p-value oLVSD vs sDD	p-value oLVSD vs control	p-value sDD vs control
LVOT obstruction† at follow-up visit, n (%)	0 (0%)	28 (39.4%)	30 (37.5%)	**0.001**	**0.002**	**0.002**	0.939
LVOT gradient‡ follow-up (mmHg)	-	85.0 (61.5-116)	64.0 (50.2-80.0)	**0.036**	-	-	0.234
Septal s′ wave follow-up (cm/s)	4.0 (3.0-4.5)	5.0 (4.0-6.0)	6.0 (5.3-7.7)	**<0.001**	**0.027**	**<0.001**	**<0.001**
Lateral s′ wave follow-up (cm/s)	4.6 (1.3)	5.7 (1.5)	7.2 (1.8)	**<0.001**	0.078	**<0.001**	**<0.001**
LAESV follow-up (ml)	158 (113-172)	121 (88.0-150)	79.0 (64.0-100)	**<0.001**	0.068	**<0.001**	**<0.001**
LAESVi follow-up (ml/m2)	80.5 (56.5-94.0)	62.2 (49.9-81.5)	41.0 (34.5-51.5)	**<0.001**	0.113	**<0.001**	**<0.001**
E/A ratio follow-up	1.0 (0.6-2.6)	1.2 (0.8-1.9)	0.9 (0.7-1.2)	**0.009**	0.716	0.716	**0.017**
E/e′ ratio follow-up	12.7 (8.2-15.3)	17.6 (14.3-22.2)	9.4 (7.7-11.0)	**<0.001**	**0.001**	**0.043**	**<0.001**
RVMWT follow-up (mm)	7.0 (6.9-8.3)	7.0 (5.6-8.0)	6.0 (5.0-7.0)	**0.038**	0.114	**0.048**	0.362
RV s′ wave follow-up	7.7 (7.0-8.4)	12.0 (10.0-13.0)	13.0 (11.0-13.8)	**<0.001**	**<0.001**	**<0.001**	**0.034**
Severity of MR at follow-up visit:				-	0.972	0.071	**0.003**
No regurgitation	0 (0%)	1 (1.4%)	6 (7.5%)				
Mild regurgitation	11 (47.8%)	33 (46.5%)	53 (66.2%)				
Moderate regurgitation	7 (30.4%)	18 (25.4%)	16 (20.0%)				

Variable	End-stage HCM with oLVSD (N=23)	End-stage HCM with sDD (N=71)	Control HCM (N=80)	p-value overall	p-value oLVSD vs sDD	p-value oLVSD vs control	p-value sDD vs control
Moderate to severe regurgitation	4 (17.4%)	14 (19.7%)	5 (6.25%)				
Severe regurgitation	1 (4.4%)	5 (7.0%)	0 (0%)				
Severity of TR at follow-up visit:				**0.008**	0.081	**0.045**	0.051
No regurgitation	0 (0%)	6 (8.5%)	4 (5.0%)				
Mild regurgitation	14 (63.6%)	43 (60.6%)	65 (81.2%)				
Moderate regurgitation	3 (13.6%)	18 (25.4%)	9 (11.2%)				
Severe regurgitation	3 (13.6%)	2 (2.8%)	2 (2.5%)				
Massive/ torrential regurgitation	2 (9.1%)	2 (2.8%)	0 (0.%)				
**Clinical findings at follow-up visit:**							
Symptoms at follow-up visit, n (%)	21 (91.3%)	65 (91.5%)	60 (75.0%)	**0.015**	1.000	0.221	**0.040**
Dyspnea at follow-up visit, n (%)	22 (95.7%)	60 (84.5%)	51 (63.7%)	**0.001**	0.282	**0.010**	**0.010**
Dyspnea NYHA≥III, n (%)	17 (73.9%)	19 (26.8%)	5 (6.3%)	**<0.001**	**<0.001**	**<0.001**	**0.001**
Palpitations at follow-up visit, n (%)	5 (21.7%)	12 (16.9%)	13 (16.2%)	0.819	1.000	1.000	1.000

Variable	End-stage HCM with oLVSD (N=23)	End-stage HCM with sDD (N=71)	Control HCM (N=80)	p-value overall	p-value oLVSD vs sDD	p-value oLVSD vs control	p-value sDD vs control
Syncope at follow-up visit, n (%)	4 (17.4%)	9 (12.7%)	9 (11.2%)	0.699	0.985	0.985	0.985
Angina at follow-up visit, n (%)	1 (4.35%)	16 (22.5%)	25 (31.2%)	**0.027**	0.093	0.057	0.308
HCM risk score at follow-up visit (%)	2.9 (2.3-6.1)	3.7 (2.4-5.8)	2.4 (1.6-4.3)	**0.020**	0.725	0.165	**0.022**
**Biomarkers at follow-up visit:**							
BNP at follow-up visit (pg/ml)	429 (222-1246)	310 (181-604)	177 (80.1-358)	**0.028**	0.501	0.185	**0.033**
NT-proBNP at follow-up visit (pg/ml)	6464 (2695-18276)	2690 (1620-4888)	892 (575-1096)	**<0.001**	0.180	**0.002**	**0.002**
**Management and treatment:**							
ICD, n (%)	9 (39.1%)	16 (22.5%)	11 (13.8%)	**0.031**	0.233	**0.042**	0.233
Pacemaker, n (%):	5 (21.7%)	6 (8.5%)	5 (6.3%)	0.079	0.195	0.126	0.837
CRT-P, n (%)	1 (4.4%)	1 (1.4%)	0 (0%)	0.127	0.473	0.473	0.473
CRT-D, n (%)	2 (8.7%)	0 (0%)	0 (0%)	**0.017**	0.058	0.058	-
Septal myectomy, n (%)	0 (0%)	5 (7.0%)	2 (2.5%)	0.297	0.494	1.000	0.494
Alcohol septal ablation, n (%)	0 (0%)	1 (1.4%)	3 (3.8%)	0.787	1.000	1.000	1.000
ACEI or sartans, n (%)	13 (56.5%)	37 (52.1%)	46 (57.5%)	0.794	1.000	1.000	1.000

Variable	End-stage HCM with oLVSD (N=23)	End-stage HCM with sDD (N=71)	Control HCM (N=80)	p-value overall	p-value oLVSD vs sDD	p-value oLVSD vs control	p-value sDD vs control
Mineralocorticoid receptor antagonist, n (%)	17 (73.9%)	24 (33.8%)	17 (21.2%)	**<0.001**	**0.003**	**<0.001**	0.122
Loop diuretic, n (%)	17 (73.9%)	36 (50.7%)	18 (22.5%)	**<0.001**	0.087	**<0.001**	**0.001**
Beta-blocker, n (%)	20 (87.0%)	64 (90.1%)	74 (92.5%)	0.582	0.822	0.822	0.822
Nondihydropyridine calcium channel blocker, n (%)	2 (8.7%)	6 (8.5%)	1 (1.3%)	0.059	1.000	0.187	0.155
**Outcomes:**							
All cause death, n (%)	10 (43.5%)	15 (21.1%)	5 (6.3%)	**<0.001**	0.066	**<0.001**	**0.021**

*Defined by more than 20% of all QRS complexes on standard 24-hour Holter monitoring.

†Defined by LVOT gradient at rest ≥30 mmHg.

‡Considering only obstructive cases, excluding those with gradients below 30 mmHg.

Abbreviations: ACEI: angiotensin-converting-enzyme inhibitors; ATP: anti-tachycardia pacing; BNP: brain natriuretic peptide; CMR: cardiovascular magnetic resonance; CRT-D: cardiac resynchronization therapy defibrillator; CRT-P: cardiac resynchronization therapy pacemaker; HCM: hypertrophic cardiomyopathy; ICD: implantable cardioverter-defibrillators; IVS: interventricular septum; L: likely pathogenic; LAESV: left atrial end-systolic volume; LAESVi: indexed left atrial end-systolic volume; LBBB: left bundle branch block; LGE: late gadolinium enhancement; LVEDDi: indexed left ventricular end-diastolic diameter; LVEDVi: indexed left ventricular end-diastolic volume; LVEF: left ventricular ejection fraction; LVESVi: indexed left ventricular end-systolic volume; LVMWT: left ventricular maximum wall thickness; LVOT: left ventricular outflow tract; MR: mitral regurgitation; NSVT: nonsustained ventricular tachycardia; NT-proBNP: N-terminal pro-brain natriuretic peptide; NYHA: New York Heart Association; oLVSD: overt left ventricular systolic dysfunction; P: pathogenic; PVCs: premature ventricular contractions; RBBB: right bundle branch block; PW: posterior wall; RVMWT: right ventricular maximum wall thickness; SCD: sudden cardiac death; sDD: severe diastolic dysfunction; SVT: sustained ventricular tachycardia; TR: tricuspid regurgitation; VT: ventricular tachycardia; VUS: variant of unknown significance.
